# Response of Soybean to Hydrochar-Based *Rhizobium* Inoculation in Loamy Sandy Soil

**DOI:** 10.3390/microorganisms8111674

**Published:** 2020-10-28

**Authors:** Dilfuza Egamberdieva, Hua Ma, Jakhongir Alimov, Moritz Reckling, Stephan Wirth, Sonoko Dorothea Bellingrath-Kimura

**Affiliations:** 1 Leibniz Centre for Agricultural Landscape Research (ZALF), Eberswalder Str. 84, 15374 Müncheberg, Germany; mh3660344@126.com (H.M.); Moritz.Reckling@zalf.de (M.R.); Stephan.Wirth@zalf.de (S.W.); Sonoko.Bellingrath-Kimura@zalf.de (S.D.B.-K.); 2 Faculty of Biology, National University of Uzbekistan, Tashkent 100174, Uzbekistan; jahongir.alimov@gmail.com; 3 School of Life Sciences, Chongqing University, Chongqing 401331, China

**Keywords:** *Bradyrhizobium japonicum*, soybean, rainfed, nodulation

## Abstract

Hydrochar is rich in nutrients and may provide a favorable habitat or shelter for bacterial proliferation and survival. Therefore, in this study, we investigate the efficiency of a hydrochar-based rhizobial inoculant (*Bradyrhizobium japonicum*) on the symbiotic performance of soybean under both greenhouse and field conditions. There were positive and significant effects of hydrochar-based inoculation on the root and shoot growth of soybean as compared to uninoculated plants grown under irrigated and drought conditions. The drought stress significantly inhibited the symbiotic performance of rhizobia with soybean. Soybean inoculated with hydrochar-based *B. japonicum* produced twofold more nodules under drought stress conditions as compared to plants inoculated with a commercial preparation/inoculant carrier *B. japonicum* (HISTICK). The N concentration of inoculated plants with hydrochar-based *B. japonicum* was by 31% higher than that of un-inoculated plants grown in pots and by 22% for HISTICK. Furthermore, the soybean treated with hydrochar-based *B. japonicum* showed higher grain yield of 29% under irrigated conditions and 40% higher under rainfed condition compared to un-inoculated plants. In conclusion, the obtained results proved the potential of hydrochar-based *B. japonicum* inoculant for soybean in terms of increased symbiotic performance and agronomic traits, especially under rainfed conditions.

## 1. Introduction

Soybean is an important legume and source of food, oil, and as a forage crop, is widely grown in China and other countries of the world [1,2]. Legumes, associated with rhizobia, can fix nitrogen, increase the availability of nitrogen in several agroecosystems. According to previous reports, half of all N (4–5 million t) supplied in agricultural systems was fixed by legumes, and it can be increased by improving the soybean-*Rhizobium* symbioses [3,4]. Therefore, plant-microbe interaction, especially in drought-affected regions, may have an essential role in maintaining highly productive legume cultivation. Soybean establishes a symbiotic association with *Bradyrhizobium japonicum*, where bacteria use nutrients and carbohydrates provided by the plant and fix atmospheric nitrogen and make it bioavailable to the plant [5]. Soybean may fix up to 50% N via its symbiosis with *Bradyrhizobum japonicum* present in the soil or applied as inoculants [6,7,8].

Several studies reported that abiotic factors, including drought and high temperature, might cause nodulation failure, because of the low survival of introduced bacteria in the soil and root system [9,10,11]. Furthermore, high competition for nutrients and niches occurs among microbes, including *Rhizobia* in the rhizosphere of plants in arid lands, which affects the bacterial ability to form nodules.

The formulation of microbes with various carrier substrates has been considered as an improvement in rhizobial survival and symbiotic effectiveness under different environmental conditions [12,13,14]. However, the short shelf life in the formulated substrate and low rhizobial survival after seed coating because of desiccation are key complications [15]. Under drought condition, a decreased survival of peat-based rhizobia inoculants in soil and failure of symbiotic performance with the host was observed [16].

Peat is considered as a carrier for the growth and survival of *Rhizobia* and widely used for field applications [17,18]. Other reports confirm perlite as a successful carrier for *B. japonicum* applied for soybean in the greenhouse and under field conditions [19,20]. Albareda et al. [21] suggested perlite as an alternative to peat for soybeans inoculants as it supports better survival of bacteria than peat. Furthermore, the shortage of peat deposits in many countries lead to the search for alternative carrier materials for rhizobial inoculants [21]. Hydrochar is another material to carry *Rhizobium*, produced via hydrothermal carbonization (HTC) at a temperature range of 180–260 °C during which biomass is heated in water in a closed system [22]. Hydrochar proved to be suitable as a bacterial carrier since it contains soluble organic compounds combined with additional habitat niches for microbial colonization [23]. In addition, several reports indicate a positive impact of hydrochar application on soil cation exchange capacity [24,25,26], water holding capacity [27], soil organic matter content, and plant growth [28,29]. Moreover, hydrochar improved soil fertility by increasing soil nutrient contents and providing increased amounts of available nutrients [30]. Hence, the selection of carriers suitable for bacterial inoculants must consider several factors, such as cost-effectiveness, non-toxic properties for microbes and the environment, and should be generally available, especially in countries that lack natural peat deposits [21]. In the areas where soybean is not naturally present or a newly introduced crop, such as Central Europe, legumes require inoculation with effective strains of *Rhizobia* [31]. The presence and survival of *Rhizobia* in soil are usually dependent on the presence of their legume host. Therefore, farmers are advised to apply rhizobial inoculants to benefit fully from biological nitrogen fixation. Given the increasing importance of soybean production in Europe, especially under drought or in semi-arid regions, the development and testing of rhizobial formulations combined with a survey of the agronomic performance under rainfed conditions would be promising. The aim of this study was to; (i) evaluate hydrochar produced from maize silage for its suitability as a carrier of *Bradyrhizobium japonicum*, and (ii) test the effectiveness of hydrochar-based *B. japonicum* inoculants forming root nodules with soybean, and (iii) survey improved growth, nutrient uptake and yield under both greenhouse and field conditions.

## 2. Materials and Methods

### 2.1. Soil, Plant, Hydrochar and Rhizobia

The soil used for pot experiments was from an arable field under irrigation operated by the Experimental Field Station of the Leibniz Centre for Agricultural Landscape Research (ZALF), Müncheberg, Germany. Selected chemical and physical properties of soil are as follows: clay and fine silt, 7%; coarse and medium silt, 19%; sand, 74%; Corg, 570 mg 100 g^−1^; pH 6.2; organic C content, 0.55%; total N content, 0.07%; P content, 32.0 mg (100 g soil)^−1^; K content, 1.25 g (100 g soil)^−1^; and Mg content, 0.18 g (100 g soil)^−1^ [32].

The soybean (*Glycine max* (L.) Merr.) cultivar Sultana was used for the pot and field experiments. The hydrochar was obtained from the Leibniz-Institute for Agrartechnik Potsdam-Bornim e.V. (ATB), and used as carrier material for bacteria. The hydrochar was produced from maize silage by batch-wise hydrothermal carbonization at 210 °C and 23 bar for 8 h and had the following properties: C, 64.6%, N, 2.1%, P, 1.0%, K, 3.6%, pH 5.3).

The strain *Bradyrhizobium japonicum* (HAMBI 2314) was obtained from the Culture Collection of the University of Helsinki (HAMBI). The strain was grown on yeast extract-mannitol (YEM) medium at 28 °C. As a control, the formulated strain *Bradyrhizobium japonicum* (HISTICK^®^ Soy, BASF Services Europe Inc., Limburgerhof, Germany) was used at a rate of 2 × 10^9^ CFU/g for the inoculation of soybean.

### 2.2. Survival of Bradyrhizobium japonicum in HTC Char

For the preparation of the bacterial inoculum, hydrochar was powdered, filled in glass tubes and sterilized at 121 °C for 20 min. The bacterial strain *Bradyrhizobium japonicum* (HAMBI 2314) was grown in yeast extract mannitol (YEM) broth. After three days, bacterial cells were washed with phosphate-buffered saline and adjusted to 10^7^ CFU mL^−1^. The bacterial cell suspension (5 mL) was uniformly and aseptically mixed with 10 g of sterilized HTC powder. The glass tubes were incubated at 28 °C for eight weeks, and every two weeks, samples were taken to analyze inoculum survival. One g of HTC-based inoculum was serially diluted (10-fold) in PBS and plated in triplicate on YEM agar plates supplemented with Congo red. The mean values of the viable bacteria (CFU) per g of the samples were determined.

### 2.3. Pot Experiments

To prepare hydrochar-based inocula for coating seeds, *B. japonicum* HAMBI2314 was grown in YEM broth for three days and adjusted to a final concentration of approximately 10^8^ CFU mL^−1^. Then, 50 mL of bacterial cell suspension was mixed with 10 g HTC and incubated for one week at 28 °C to facilitate bacterial growth. Soybean (*Glycine max* L. var. *sultana*) seeds surface-sterilized using 10% *v/v* NaOCl for 5 min and 70% ethanol for 5 min, and then rinsed five times with sterile distilled water. Finally, surface-sterilized seeds were coated with bacterial inoculants. The inoculation treatments were as follows: i) uninoculated seeds, ii) seeds inoculated with HTC-based inoculum of *B. japonicum* (BHTC), and iii) seeds coated with *B. japonicum* (HISTICK). Six replicate pots were used per treatment (*n* = 6). All pots were arranged in a randomized block design. The plants were grown in a greenhouse (day/night temperature 25 °C/18 °C; humidity 50–60%, day length 12 h for 35 days. Two conditions, namely watered (at 75% field capacity) and drought stress (watered at 45% field capacity) were maintained by daily weighing and watering each pot if required. At harvest, the roots were separated from shoots and were oven-dried at 70 °C for 48 h, and dry weight was determined. The number of nodules (nodule size >1 mm) was visually counted for each plant.

### 2.4. Analysis of Plant Nutrients

Nitrogen (N), phosphorus (P), and potassium (K) contents in plant tissue were determined with an inductively coupled plasma optical emission spectrometer (ICP-OES; iCAP 6300 Duo, Termo Fisher Scientific Inc., Watham, MA, USA).

### 2.5. Plant Growth Under Field Conditions

The field experiment was conducted at the Experimental Field Station of the Leibniz Centre for Agricultural Landscape Research (ZALF), Müncheberg, Germany (52°31’17.4″N 14°07’40.0″E). A total of 12 plots of 18 m^2^ with a harvesting area of 11.75 m^2^ were established. The experiment was conducted in a complete block design with three replications. Four treatments were randomly arranged; the treatments were: (a) No bacterial inoculation, with irrigation, (b) no bacterial inoculation, without irrigation, (c) inoculation with *B. japonicum* (BHTC), with irrigation, and (d) inoculation with *B. japonicum* (BHTC), without irrigation.

Soybean was cultivated using regular machinery according to local farming practices. Sowing was done by a drilling machine on 10 April 2015; no fertilizers or herbicides were applied. Harvesting was conducted on 5 October 2015 using a harvester combined with a yield-measuring system. Precipitation and temperature were collected on a daily basis by the weather station of ZALF. The year 2015 was exceptionally dry with a mean precipitation of 473 mm as compared to the 30-year mean of 555 mm with water shortages during the entire soybean growth period. The mean temperature was 10.3 °C higher as compared to the 30-year mean of 9.1 °C. Irrigation water was applied with a sprinkler system using the Web-BEREST model to determine the amounts and timing as described in Reckling et al. [33]. In 2015, 165 mm of irrigation water were applied at ten dates (10–25 mm per date).

Soybean was planted in eight rows for each plot, the row space was 50 cm, and the seeding rate was 80 seeds m^−2^. Seeds were inoculated with hydrochar-based *B. japonicum* HAMBI2314 at a rate of 7 × 10^8^ CFU/g before planting. After 90 days, ten plants were sampled from each plot, and then the roots were washed with tap water, and nodule numbers were determined. The plant biomass (30 July 2015) and grain (5 October 2015) were harvested plot by plot, and yields were estimated.

### 2.6. Statistical Analyses

All data were performed to the general linear model procedure to analyze the variance. The significance between treatments was tested by Duncan test at the *p* < 0.05 level. The interactions between factors were conducted by general linear model. All these analyses were performed with SPSS 22 (IBM Deutschland GmbH, Ehningen, Germany).

## 3. Results

### 3.1. Survival of B. japonicum in the HTC Carrier

Survival and viability of *B. japonicum* HAMBI2314 over eight weeks in the HTC-char carrier materials showed that hydrochar is a suitable carrier for the bacterial inoculant. After two weeks, the log10 CFU count of *B. japonicum* HAMBI2314 was 5.1 ± 0.11. Only after six and eight weeks, there were decreases in the bacterial populations being 4.6 ± 0.17, and 4.2 ± 0.14 Log CFU, respectively.

### 3.2. Plant Growth under Greenhouse Conditions

**Figure 1:**
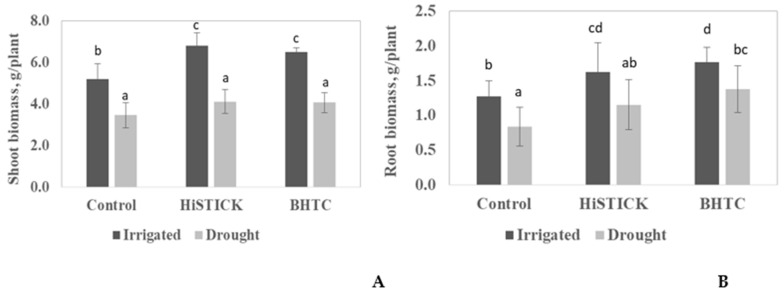
Effect of *B. japonicum* (HISTICK) and *B. japonicum* (BHTC, HTC- based rhizobia) inoculants on soybean shoot; (**A**) and root; (**B**) dry weight under watered and drought conditions. The plants were grown in a greenhouse for 35 days. Column means marked by a different letters indicate significant differences at the *p* < 0.05.

The root and shoot of soybean responded differently to HISTICK and *B. japonicum* (BHTC) inoculants under-watered and drought conditions (Figure 1). The growth of soybean shoots inoculated with HISTICK was significantly higher (*p* < 0.05) than the un-inoculated plants. Furthermore, there were positive and significant effects of BHTC inoculations on the root and shoot growth of soybean grown under watered conditions. The shoot growth was significantly increased by 25% and root growth by 39% when compared to un-inoculated control plants (Figure 1A,B).

**Figure 2:**
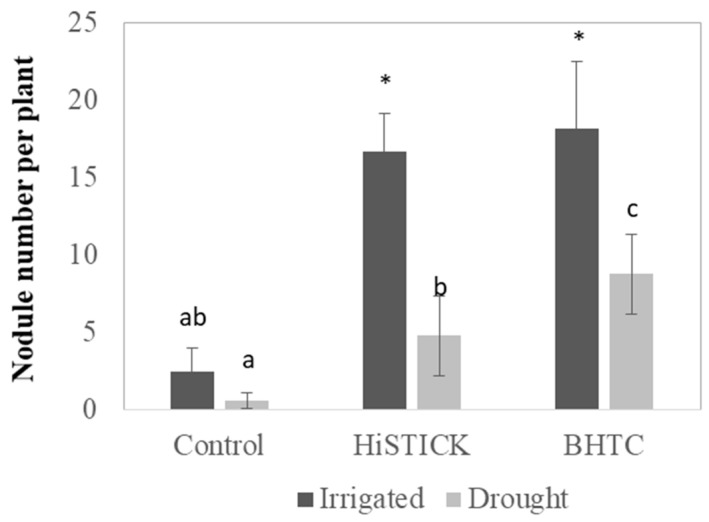
Nodule numbers of soybean under watered and drought conditions in a pot experiment. Plants were inoculated with B. japonicum (HISTICK) and B. japonicum (BHTC, HTC-based Rhizobia). The plants were grown in a greenhouse for 35 days. Column means marked by a different letters indicate significant differences at the * *p* < 0.05.

The drought stress negatively affected the root and shoot growth of soybean. The weight of shoots and roots were reduced by 33% and 34%, respectively (Figure 1). The soybean growth responded differently after bacterial inoculation with HISTICK or BHTC. The inoculation of soybean with HISTICK enhanced root and shoot growth by 19 and 25% compared to un-inoculated plants, respectively. The BHTC inoculant improved the root growth of soybean even more than the HISTICK inoculant under drought conditions, being significantly higher by 36% as compared to un-inoculated control plants. The drought stress significantly inhibited the symbiotic performance of rhizobia with soybean. However, soybean inoculated with BHTC produced twofold more nodules under drought stress conditions as compared to plants inoculated with HISTICK (Figure 2).

The inoculation of soybean with HISTICK and BHTC positively affected on N, P, and K contents of plants (Table 1). The N concentration of inoculated plants with BHTC was by 31% higher than that of un-inoculated plants and by 22% for HISTICK. There were slight increases of P and K uptake by inoculation of soybean, however, not significant increases. The acquisition of N by soybean was significantly improved by BHTC inoculation under drought conditions, but there were no significant effects on P and K uptake of plants by both inoculants (Table 1). Treatments × irrigation showed a significant interaction effect on the nodule number, but no interaction effect on N, P, K content, or shoot and root biomass (Table 2).

### 3.3. Soybean Growth under Field Conditions

**Figure 3:**
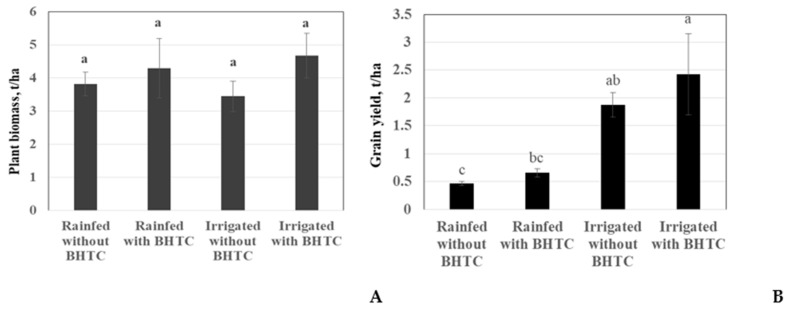
The effect of hydrochar-based inoculant *B. japonicum* (BHTC) on plant biomass; (**A**) and grain yield; (**B**) of soybean under field conditions (tons per hectare). Column means marked by a different letters indicate significant differences at the *p* < 0.05.

In previous pot experiments, the BHTC inoculant showed good performance in terms of nodulation, and was thus, used for further field trials. The effects of bacterial inoculants on soybean growth and yield under irrigated and rainfed conditions are shown in Figure 3 A,B. The plant biomass was affected by the bacterial inoculant and also by irrigation. At the same time, the average of irrigated treatments provided 3.5 and 4.6 t ha^−1^ plant biomass for the un-inoculated and the BHTC variants, respectively. Compared with un-inoculated plants, the plant biomass inoculated with BHTC was increased by 12% under rainfed condition. However, the difference was not significant (Figure 3A).

**Figure 4:**
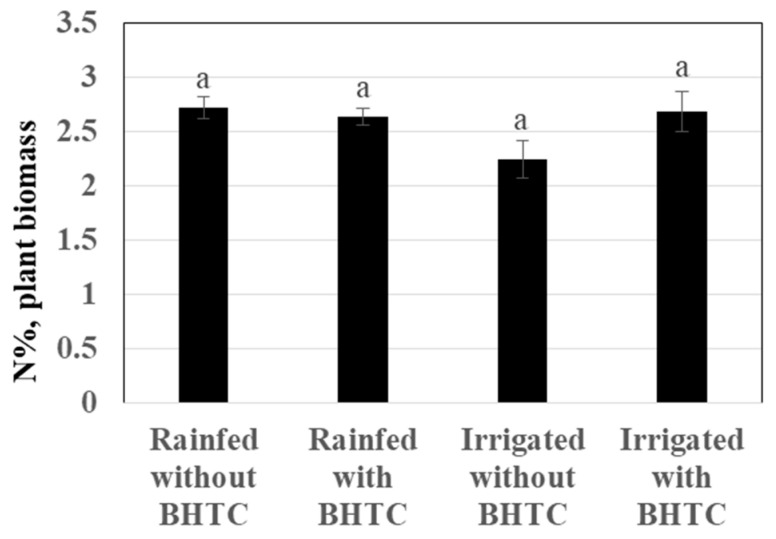
The effect of hydrochar-based inoculant *B. japonicum* (BHTC) on N% in plant biomass under field conditions. Column means marked by letters indicate significant differences at the *p* < 0.05.

Furthermore, the soybean treated with BHTC inoculant showed higher grain yield (29% and the absolute difference was 0.55 t/ha) than the uninoculated control plants in irrigated plots (Figure 3B). The grain yield of soybean was reduced under the rainfed condition by three-fold. The grain yield of soybean treated with BHTC was higher (40% and the absolute difference was 0.19 t/ha) than for un-inoculated plants under rainfed condition. The N contents of plants inoculated with BHTC were increased under irrigation only. The irrigated plots with BHTC showed 19% (0.4% N) higher values for N contents in the shoot than the plots without BHTC (Figure 4).

## 4. Discussion

For successful bacterial inoculation, it is essential to use a substrate that holds the potential as a carrier for microbes, which supports microbial shelf life, protects from stressors, and contributes as a nutrient source for propagation [15,34]. The porosity and a low ash content of biochar are essential factors for the growth of beneficial microbes that determine the extent of bacterial adhesion, cohesion, and proliferation. The present work focusses on the analysis of hydrochar-based *B. japonicum* for its symbiotic performance with soybean and plant growth under rainfed conditions. The bacterial inoculants based on hydrochar as carrier was demonstrated to promote plant growth, seed yield, and nutrient acquisition [14,35,36]. For example, Saxena et al. [37] observed a positive effect of combined inoculation of *Bacillus* sp. with hydrochar on the plant biomass, pod formation and seed yield of common bean (*Phaseolus vulgaris*). In our study, biochar-based *B. japonicum* HAMBI2314 improved soybean root and shoot growth and nutrient uptake under both irrigated and drought conditions as compared to un-inoculated control plants. The significantly higher nodule numbers explain the improved N concentrations in plant tissue. The hydrochar-based *B. japonicum* inoculant improved soybean growth under drought conditions, which are even better than a commercial inoculant, hence, confirming the agronomic potential of hydrochar-based inocula is important.

We observed that the inoculant *B. japonicum* HAMBI2314 survived in hydrochar for six and eight weeks after incubation, which proves the suitability of this material as an inoculant carrier. Similar observations were demonstrated by Khavazi et al. [38] when *B. japonicum* could survive in biochar pores for more than six months. Tripti et al. [36] also observed higher CFU of *Bacillus* sp. and *Burkholderia* sp. in a biochar substrate after 240 days of storage. In an earlier report by Głodowska et al. [39], the effective survival of *B. japonicum* in Dynamotive and Pyrovac biochars was reported. The hydrochar showed a similar porous structure, compared to biochar, which supports microbial proliferation by providing nutrients, water and protection from abiotic stresses [40,41]. Moreover, it contains higher amounts of soluble organic compounds used by microorganism as C resource. According to Li et al. [42], biochar provides cationic and neutral surface properties and contains functional groups such as carboxyl and hydroxyl groups, allowing rapid immobilization of bacterial cells. Thus, biochar might be a promising effective carrier for beneficial inoculants.

In the current study, soybean seeds inoculated with a hydrochar-based inoculant of *B. japonicum* HAMBI2314 showed increased nodule numbers. Improved symbiotic performance of biochar-formulated *B. japonicum* was also reported by Glodowska et al. [41]. In another study, Ghazi [43] demonstrated an enhanced nodule formation in kidney bean by biochar-based *Rhizobia*. The finding of Sangeetha [44,45] indicates that biochar pores play an essential role in bacterial proliferation, by improving gas exchange, nutrient and water supply and protection from environmental stresses. Once the application of BHTC inoculants showed improved growth and symbiotic performance with soybean in pot experiments, the next step was to validate the optimal formulation to be applied as an inoculant under field conditions. In our field trials, BHTC inocula increased plant biomass, grain yield and N uptake of soybean both under irrigated and rainfed conditions. Similar observations were reported for lupin when hydrochar-based *Bradyrhizobium* sp. increased plant growth, pod formation and grain yield under both irrigated and rainfed conditions compared to un-inoculated control plants [32]. Saxena et al. [37] observed an increase of plant biomass and yield of the common bean by a combined application of biochar with *Bacillus* sp. However, even if an efficient link between symbiotic performance, N concentration and plant growth can be assumed, the effects were often small and not significant under field conditions due to additional constraints in crop growth. Nevertheless, the improvement of soybean yield by hydrochar-based rhizobial inoculants under rainfed field conditions suggests that hydrochar as a formulation or carrier plays an essential role in the symbiotic efficiency of rhizobia in soybean.

## 5. Conclusions

In conclusion, the obtained results proved the potential of the hydrochar-based *B. japonicum* inoculant for soybean in terms of increased symbiotic performance and agronomic traits, especially under rainfed conditions. This study indicated that hydrochar-based rhizobial inoculants could contribute to a step-change in the sustainable cu ltivation of soybean, especially at local dry conditions and act as an alternative to other commercially used materials, such as peat-derived materials.

## Figures and Tables

**Table 1:** Nutrient concentrations in soybean inoculated with B. japonicum (HISTICK) and B. japonicum (BHTC, HTC-based Rhizobia) under watered and drought conditions. The plants were grown in a greenhouse for 35 days.

	Watered			Drought		
	N	P	K	N	P	K
**Control**	2.263c	0.271a	1.920b	1.803a	0.254a	1.459a
**HISTICK**	2.782d	0.277a	2.086b	1.923ab	0.270a	1.511a
**BHTC**	2.983d	0.278a	1.954b	2.149bc	0.285a	1.502a

**Table 2:** Interaction effects of treatments (Control, HISTICK, BHTC), and irrigation (Watered, Drought) on the plant N, P, K content, shoot and root biomass, and nodule number. Significance denoted by *** *p* < 0.001.

Greenhouse	N	P	K	Shoot	Root	Nodule
Treatments × Irrigation	ns	ns	ns	ns	ns	***
